# Association between duration of Untreated Psychosis and severer symptoms and poorer quality of life: study in First-Episode Psychosis patients

**DOI:** 10.1192/j.eurpsy.2023.2216

**Published:** 2023-07-19

**Authors:** A. C. M. Pires, A. Araújo, S. Ramos Ferreira, A. Bajouco, M. Bajouco

**Affiliations:** 1 Psychiatry, Coimbra Hospital and Universitary Centre; 2 Institute of Psychological Medicine, Faculty of Medicine of the University of Coimbra; 3 Coimbra Institute for Biomedical Imaging and Translational Research, University of Coimbra, Coimbra, Portugal

## Abstract

**Introduction:**

The First Episode Psychosis (FEP) Inpatient Unit is specialized in early assessment/intervention in patients with Psychotic Disorders. Duration of Untreated Psychosis/DUP has a key role in the prognosis of those patients. Longer DUP is associated with poorer treatment response and greater risk of relapse. Some studies also suggest an association between DUP and the severity of negative symptoms, but further research is needed.

**Objectives:**

The objectives of this study were to analyze the relationship between DUP and psychotic symptoms, duration of admission, medication, and quality of life, after inpatient intervention.

**Methods:**

This is a retrospective study of a cohort (N=25) admitted to the unit. Sociodemographic and clinical variables (number of days of hospitalization, DUP, and Defined Daily Dose/DDD of antipsychotics) were evaluated. Psychometric instruments (PANSS/Positive and Negative Syndrome Scale and WHOQOL-BREF/World Health Organization Quality Of Life) were applied at admission and at discharge. We used a Spearman correlation test to measure the degree of association between the variables.

**Results:**

Longer DUP correlated with more days of hospitalization, higher negative PANSS scores, and poorer social relationships domain of the WHOQOL at admission (p<0.05). At discharge, DUP presented positive and significant correlations with all subscales of the PANSS (positive, negative, and general; p<0.05) and DDD (p<0.01).

**Table:** 

**VARIABLES**	**DUP**
**WHOQOL_SOCIAL RELATIONSHIPS DOMAIN_ADMISSION**	r_s_ =-.448* p=0.018
**PANSS_NEGATIVE_ ADMISSION**	r_s_ =.424* p=0.035
**PANSS_NEGATIVE_ DISCHARGE**	r_s_ =.638** p=0.001
**PANSS_POSITIVE_ DISCHARGE**	r_s_ =.455* p=0.022
**PANSS_GENERAL_ DISCHARGE**	r_s_ =.518** p=0.008
**PANSS_TOTAL_ DISCHARGE**	r_s_ =.564** p=0.003
**DDD_ DISCHARGE**	r_s_=.539** p=0.005
**DAYS OF HOSPITALIZATION**	r_s_=.429** p=0.032

**Conclusions:**

Our results are in line with the current literature on DUP, showing it leads to a worse prognosis, with a more severe clinical course, with the need for extended hospitalizations, a worsening of social relationships, and a higher dosage of medication.

Thus, DUP may be a potentially modifiable prognostic factor. It is possible that FEP patients with negative symptoms dominance may have a more insidious onset and, therefore, the search for treatment may be delayed. Conversely, if there is a mechanism by which DUP influences the symptom profile, its knowledge may lead to a better understanding of psychosis and improved treatment options.

Importantly, DUP showed stronger correlations with the severity of the clinical picture at discharge than at admission, suggesting that longer untreated psychosis may also predict poorer treatment response.

**Disclosure of Interest:**

None Declared

